# Multi-institutional Prognostic Modeling in Head and Neck Cancer: Evaluating Impact and Generalizability of Deep Learning and Radiomics

**DOI:** 10.1158/2767-9764.CRC-22-0152

**Published:** 2023-06-29

**Authors:** Michal Kazmierski, Mattea Welch, Sejin Kim, Chris McIntosh, Katrina Rey-McIntyre, Shao Hui Huang, Tirth Patel, Tony Tadic, Michael Milosevic, Fei-Fei Liu, Adam Ryczkowski, Joanna Kazmierska, Zezhong Ye, Deborah Plana, Hugo J.W.L. Aerts, Benjamin H. Kann, Scott V. Bratman, Andrew J. Hope, Benjamin Haibe-Kains

**Affiliations:** 1 Department of Medical Biophysics, University of Toronto, Toronto, Ontario, Canada.; 2 Princess Margaret Cancer Centre, Toronto, Ontario, Canada.; 3 TECHNA Institute, Toronto, Ontario, Canada.; 4 Radiation Medicine Program, Princess Margaret Cancer Centre, Toronto, Ontario, Canada.; 5 Department of Radiation Oncology, University of Toronto, Ontario, Canada.; 6 Department of Medical Physics, Greater Poland Cancer Centre, Poznan, Poland.; 7 Department of Electroradiology, University of Medical Sciences, Poznan, Poland.; 8 Department of Radiotherapy II, Greater Poland Cancer Centre, Poznan, Poland.; 9 Artificial Intelligence in Medicine (AIM) Program, Mass General Brigham, Harvard Medical School, Boston, Massachusetts.; 10 Department of Radiation Oncology, Dana-Farber Cancer Institute / Brigham and Women's Hosptial, Boston, Massachusetts.; 11 Radiology and Nuclear Medicine, CARIM and GROW, Maastricht University, Maastricht, the Netherlands.

## Abstract

Artificial intelligence (AI) and machine learning (ML) are becoming critical in developing and deploying personalized medicine and targeted clinical trials. Recent advances in ML have enabled the integration of wider ranges of data including both medical records and imaging (radiomics). However, the development of prognostic models is complex as no modeling strategy is universally superior to others and validation of developed models requires large and diverse datasets to demonstrate that prognostic models developed (regardless of method) from one dataset are applicable to other datasets both internally and externally. Using a retrospective dataset of 2,552 patients from a single institution and a strict evaluation framework that included external validation on three external patient cohorts (873 patients), we crowdsourced the development of ML models to predict overall survival in head and neck cancer (HNC) using electronic medical records (EMR) and pretreatment radiological images. To assess the relative contributions of radiomics in predicting HNC prognosis, we compared 12 different models using imaging and/or EMR data. The model with the highest accuracy used multitask learning on clinical data and tumor volume, achieving high prognostic accuracy for 2-year and lifetime survival prediction, outperforming models relying on clinical data only, engineered radiomics, or complex deep neural network architecture. However, when we attempted to extend the best performing models from this large training dataset to other institutions, we observed significant reductions in the performance of the model in those datasets, highlighting the importance of detailed population-based reporting for AI/ML model utility and stronger validation frameworks.

1. We have developed highly prognostic models for overall survival in HNC using EMRs and pretreatment radiological images based on a large, retrospective dataset of 2,552 patients from our institution.
2. Diverse ML approaches were used by independent investigators. The model with the highest accuracy used multitask learning on clinical data and tumor volume.
3. External validation of the top three performing models on three datasets (873 patients) with significant differences in the distributions of clinical and demographic variables demonstrated significant decreases in model performance.

**Significance:**

ML combined with simple prognostic factors outperformed multiple advanced CT radiomics and deep learning methods. ML models provided diverse solutions for prognosis of patients with HNC but their prognostic value is affected by differences in patient populations and require extensive validation.

## Introduction

The use of computer algorithms including artificial intelligence (AI) and machine learning (ML) to assist in clinical oncology tasks such as predicting patient prognosis (1). AI/ML has been increasingly employed when attempting to process clinical data from multiple sources and aid in diagnosis (2), prognosis (3), and course of treatment decisions (4), enabling a more precise approach to clinical management taking individual patient characteristics into account (5). The need for more personalized care is particularly evident in head and neck cancer (HNC), which exhibits significant heterogeneity in clinical presentation, tumor biology, and outcomes (6, 7), making it difficult to select the optimal management strategy for each patient. Hence, there is a current need for better prognostic tools to guide clinical decision making (8, 9).

One potential source of novel prognostic information is the imaging data collected as part of standard care. Imaging data have the potential to increase the scope of relevant prognostic factors in a noninvasive manner as compared with genomics or pathology, while high volume and intrinsic complexity render it an excellent use case for ML. *Radiomics* is an umbrella term for the emerging field of research aiming to develop new noninvasive quantitative prognostic and predictive imaging biomarkers using both hand-engineered (10) and deep learning techniques (11). In HNC, radiomics has been used to predict patient outcomes (12–14), treatment response (15, 16), toxicity (17, 18), and discover associations between imaging and genomic markers (19–21).

Despite the large number of promising retrospective studies, the adoption of prognostic models utilizing radiomics in clinical workflows is limited (22, 23). Multiple factors have affected adoption including lack of a clear superior predictive modeling strategy (24), relatively small, single-institution datasets lacking sufficient validation and generalizability, and insufficient transparency and reproducibility of ML research (25, 26). Although significant progress has been made in certain areas [e.g., ensuring consistency between different engineered feature toolkits (27)], many studies do not provide sufficient details or underlying materials (e.g., code, data) to be reproduced by other groups (22, 28). In addition, the lack of benchmark datasets makes comparing different approaches challenging. Current image quantification methods based on engineered features are also limited because of high rates of correlates and potential redundancy with accepted clinical variables and biomarkers (13, 29, 30).

To overcome these limitations, we implemented a collaborative challenge focused on reproducibility, transparency, and generalizability of radiomic modeling for HNC prognosis. Furthermore, we performed extensive external validation of models to explore how models derived from one dataset might extend to other datasets and to determine whether state-of-the-art models would be extensible. To achieve this, we used a large internal dataset of 2,552 patients with HNC and three external independent HNC patient cohorts (873 patients), multiple intrainstitutional independent investigators developed HNC prognostic models based on routine pretreatment CT imaging and data from electronic medical records (EMR). We benchmarked the 12 modeling approaches against baseline clinical and radiomics models and statistically compared their performance. The model with the highest accuracy used multitask learning on EMR data and tumor volume, outperforming more complex deep learning models. By crowdsourcing our model development and engaging external research groups for validation, we were able to demonstrate how collaborative research can be used to expedite the development of more robust radiomics models for cancer research.

## Materials and Methods

### RADCURE Prognostic Modeling Challenge

The challenge was organized by the Radiomics for Radiotherapy Research initiative at the University Health Network (radiomics.ca) and was open to anyone within the University Health Network system. The protocol describing the training and test data as well as the evaluation metrics and ranking of participants was predefined and updated on the basis of the participants’ feedback; the protocol is publicly available on GitHub (uhn-radcure-challenge). In brief, all participants had access to the training data with ground-truth outcome labels, while the test set was held out for final evaluation. The primary objective was to predict 2-year overall survival (OS), with the secondary goals of predicting a patient's lifetime risk of death and full survival curve. We chose the binary endpoint as it is commonly used in the literature and readily amenable to many standard ML methods. The evaluation of the models’ performance was primarily based on the binary endpoint, which was used to rank the submissions. We also used average precision (AP) as a secondary performance measure to break any submission ties, due to its higher sensitivity to class imbalance. Optionally, a subset of the models was also evaluated for their prognostic value, that is, risk and survival curve predictions. Importantly, the participants were blinded to test set outcomes and only submitted predictions to be evaluated by the organizers. We additionally created a set of benchmark models for comparison. We did not enforce any particular model type, image processing or input data (provided it was part of the official training set), although we did encourage participants to submit predictions based on EMR features, images, and combined data separately (if they chose to use all of the data modalities).

### Training Dataset

We collected a retrospective dataset of 2,552 patients with HNC treated with radiotherapy or combined radiotherapy and systemic therapy at Princess Margaret (PM) Cancer Centre between 2005 and 2017 (Supplementary Table S1), which we split into training and test subsets by date of diagnosis (2005–2015 and 2016–2018 for training and independent test set, respectively). The study was approved by the Institutional Research Ethics Board (#17-5871). The inclusion criteria were: (i) availability of planning CT image and target contours; (ii) at least 2 years follow-up (or death before that time); and (iii) no distant metastases at diagnosis and no prior surgery. Primary gross tumor volumes (GTV) were delineated by radiation oncologists as part of routine treatment planning. For each patient, we exported the CT image and primary GTV binary mask in NRRD format. We also extracted the follow-up information (current as of April 2020). The dataset was split into training (*n* = 1,802) and test (*n* = 750) subsets according to the date of diagnosis (Supplementary Fig. S1). The dataset was hosted on an institutional high-performance computing cluster with multicore CPUs and general-purpose graphics processing units which were available to all research partners for model training.

### Baseline Models

To provide baselines for comparison and a reference point for our collaborating partners, we created three benchmark models using: (i) standard prognostic factors used in the clinic [age, sex, T/N stage, and human papillomavirus (HPV) status] (baseline-clinical); (ii) primary tumor volume only (baseline-volume); (iii) handcrafted imaging features (baseline-radiomics). All categorical variables were one-hot encoded and missing data were handled by creating additional category representing missing value (e.g., “Not tested” for HPV status). For the baseline-radiomics model, we extracted all available first order, shape and textural features from the original image and all available filters (1,316 features in total) using the PyRadiomics package (version 2.2.0; ref. 31) and performed feature selection using maximum relevance-minimum redundancy method (32). The number of selected features and model hyperparameters (*l*_2_ regularization strength) were tuned using grid search with 5-fold cross-validation. All models were built using logistic regression for the binary endpoint and a proportional hazards model for the survival endpoint.

### Tasks and Performance Metrics

The main objective of the work was to predict binarized 2-year OS, with the supplementary task of predicting lifetime risk of death and full survival curves (in 1-month intervals from 0 to 23 months). To evaluate and compare model performance on the 2-year binarized survival prediction task, we used area under the ROC curve (AUROC), which is a ranking metric computed over all possible decision thresholds (33). We additionally computed the area under precision-recall curve, also referred to as AP, using the formula:



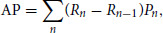



where *R_n_* and *P*_n_ are the precision and recall at a given threshold, respectively. While AUROC is insensitive to class balance, AP considers the positive class only, which can reveal pathologies under high class imbalance (34). In addition, both metrics consider all possible operating points, which removes the need to choose a particular decision threshold (which can vary depending on the downstream clinical task). Because the dataset did not include patients with follow-up time less than 2 years, we did not correct the binary metrics for censoring bias.

For the lifetime risk prediction task, we used concordance (*C*) index, defined as:



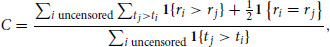



where *t*_i_ is the time until death or censoring for patient *i*, *r_i_* is the predicted risk score for patient *i*, and **1**{} is the indicator function. The agreement between the performance measures was good (Pearson *r* = 0.88 between AUROC and AP, *r* = 0.82 between AUROC and *C*-index). We compared the AUROC achieved by the best model to the other models using one-sided *t* test and corrected for multiple comparisons by controlling the FDR at 5% level.

### Independent Validation

We first assessed the performance of the prognostic models using an independent internal dataset composed of all RADCURE patients whose diagnosis took place after December 22, 2013. To further assess the generalizability of the best performing models, as well as the reproducibility of the challenge framework, we evaluated the performance of the top three models and the best deep learning model on three external datasets. The HN1 dataset is a publicly available collection^1^ of 137 patients with oropharynx and larynx tumors treated with radiochemotherapy at MAASTRO Clinic in Maastricht, the Netherlands and has been used previously in radiomics studies (35). The MDACC dataset^2^ contains data from 627 patients with oropharynx cancer treated at MD Anderson Cancer Center (36). Finally, the GPCCHN dataset is a private dataset of 298 patients treated at Greater Poland Cancer Centre in Poznan, Poland. The HN1 and MDACC datasets were obtained from the Cancer Imaging Archive (37). For all three datasets, we used the same patient selection criteria and preprocessing workflow as for the main training dataset. Validation on the HN1 dataset was performed internally within our institution. In the case of MDACC and GPCCHN datasets, we provided external collaborators (at Dana-Farber Cancer Institute and Greater Poland Cancer Centre, respectively) with code, documentation and pretrained models and asked them to compute the predictions using their own infrastructure; we were available to provide technical support if necessary. We statistically compared the variables of interest between the internal test set and each of the external test datasets using pairwise *χ*^2^ test and corrected for multiple comparisons by controlling the FDR at 5% level.

### Research Reproducibility

The code used to prepare the data, train the baseline models, evaluate the models, and analyze the results is available on Github at https://github.com/bhklab/uhn-radcure-challenge. We also share the model code for all the models in the same repository. Furthermore, we are planning to make the complete dataset, including anonymized images, contours and EMR data available on the Cancer Imaging Archive.

### Data Availability

The data used in this study are a subset of the RADCURE dataset publicly available on The Cancer Imaging Archive (37): https://doi.org/10.7937/J47W-NM11.

## Results

**FIGURE 1:**
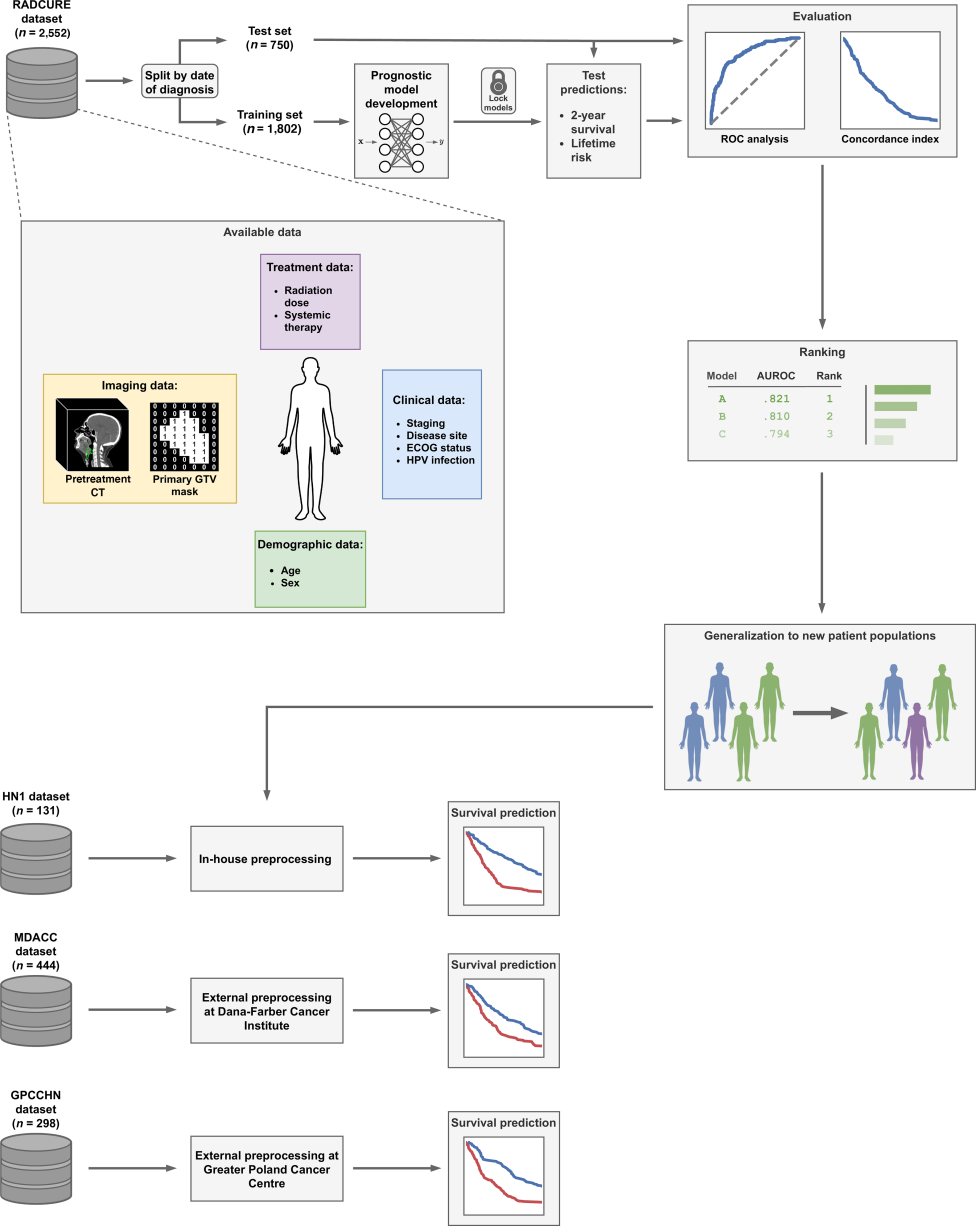
Overview of methodology and dataset. EMR and imaging data from a large cohort of patients with HNC were made available to our research partners. The training set, consisting of patients diagnosed before a prespecified date was released together with the ground-truth outcome information (OS) and used for prognostic model development. The test set was kept private and only made available (without outcome data) after the development phase was completed. We also developed a set of simple but strong baseline models to serve as benchmark for comparison as well as a reference point for our research partners during the development of their models. To assess the generalizability of the developed prognostic models to new patient populations, we performed external validation of all the models in three external datasets. Preprocessing and evaluation using the HN1 dataset was performed in-house, while evaluation using the MDACC and GPCCHN datasets was performed by external collaborators.

To assess the performance of a diverse set of prognostic modeling strategies, we have organized an institutional competition designed to leverage a large compendium of internal and external data, as well as the expertise of independent investigators using strict evaluation and validation framework (Fig. 1).

### The Challenge

The challenge was conceived and organized by the Radiomics for Radiotherapy Research initiative at the University Health Network (radiomics.ca). The protocol describing the training and test data as well as the evaluation metrics and ranking of participants was predefined, which has been made publicly available (uhn-radcure-challenge). The Challenge officially opened on April 14, 2020 with the registration closing on April 30, 2020. A total of four teams registered by the deadline and had access to the training data until July 25, 2020, which was the deadline for the final submission of their best models (each team could submit a maximum of three models). The blind evaluation of the 12 submitted models by the Challenge organizers on the independent test dataset took place until July 30, 2020. The winners were announced on July 31, 2020.

### Dataset

Given the complexity of predicting survival in patients with HNC, we collected the largest dataset to date combining EMR (i.e., clinical, demographic, and interventional data) and radiological imaging data for 2,552 patients with HNC treated with definitive radiotherapy. All the data were collected and generated within the PM Cancer Centre. The dataset was divided into a training set (70%) and an independent test set (30%) based on the date of diagnosis at a predefined timepoint (December 22, 2013). We made pretreatment contrast-enhanced CT images and binary masks of primary GTV available to our research partners. We also provided the set of available clinical variables extracted from EMR, including demographic (age at diagnosis, sex), clinical (T, N and overall stage, disease site, performance status, and HPV infection status) and treatment-related (radiation dose in Gy, use of systemic therapy) characteristics for modeling purposes (Fig. 1). In addition, outcome data (time to death or censoring, event indicator) were available for the training data only.

### Model Training and Evaluation Criteria

All research participants had access to the training data with ground-truth outcome labels (OS), while the test set was held out for final evaluation. The primary objective was to predict 2-year OS, with the secondary goals of predicting a patient's lifetime risk of death and full survival curve. We chose the binary endpoint as it is commonly used in the literature and readily amenable to many standard ML methods. The primary evaluation metric for the binary endpoint, which was used to rank the models, was the AUROC. We also used AP as a secondary performance measure to break any model ties, due to its higher sensitivity to class imbalance (38). Prediction of lifetime risk and survival curve predictions was optional, and they were scored using the *C*-index (39). Importantly, research partners were blinded to test set outcomes and only submitted predictions to be evaluated by the organizers. We additionally created a set of benchmark models using only clinical, only imaging data or a combination of both for comparison (see Materials and Methods). We did not enforce any particular model type, image processing or input data (provided it was part of the official training set), although we did encourage participants to submit predictions based on EMR features, images, and combined data separately (if they chose to use all of the data modalities).

### Overview of Models

**FIGURE 2:**
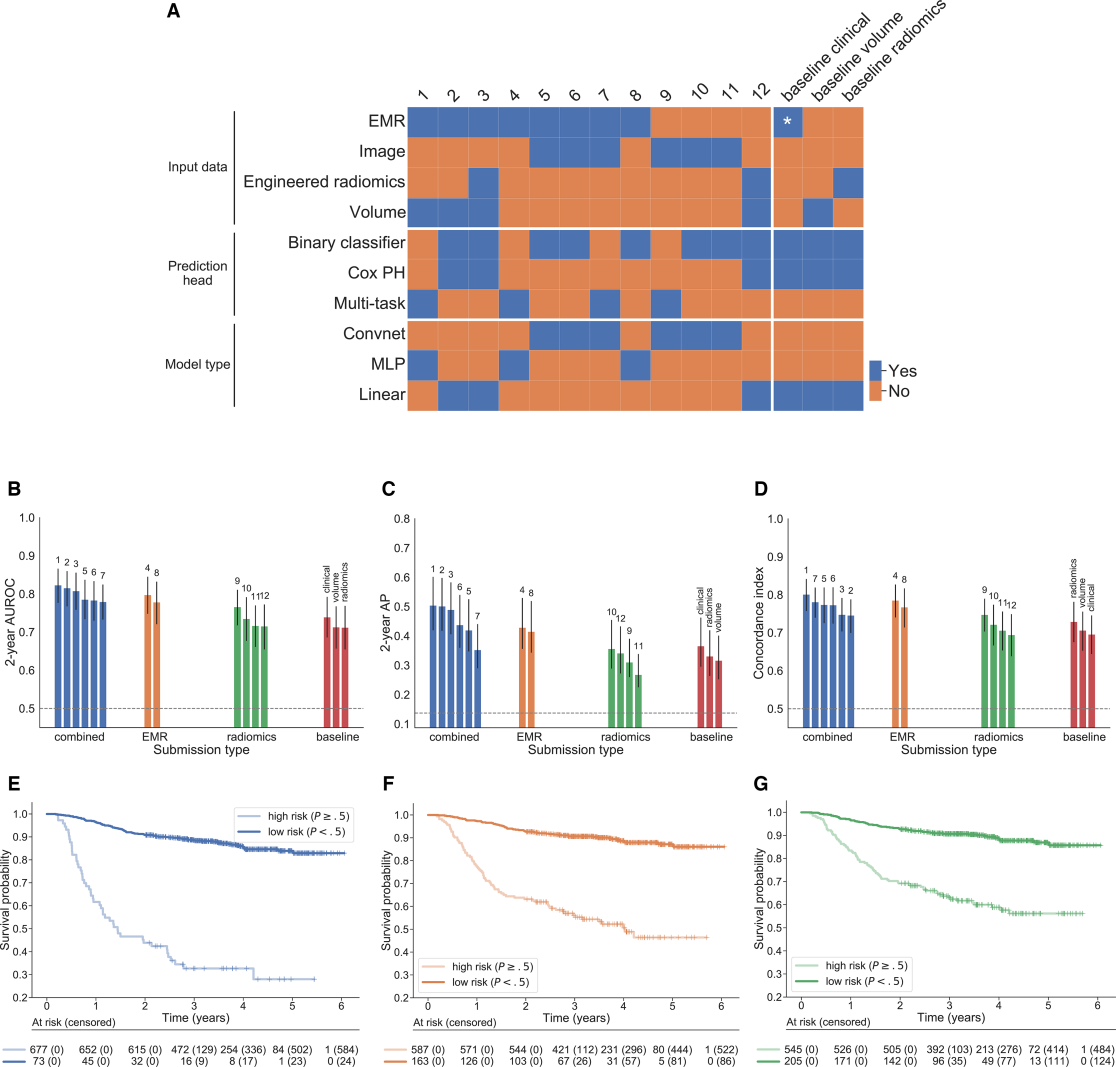
Model results. **A,** Overview of model characteristics. The characteristics are grouped into input data, prediction head (i.e., how were the survival predictions made) and model type (whether the model involved any nonlinearities and/or convolutions). PH: proportional hazards, MLP: multilayer perceptron, *: age, sex, stage, HPV status. **B–D**, Performance of all models, including benchmark models, in terms of 2-year AUROC, 2-year average precision and *C*-index of the lifetime risk, respectively. The results are ranked by AUROC (numbers above bars indicate the overall rank of each model). Error bars represent 95% confidence intervals computed using 10,000 stratified bootstrap replicates. Dashed gray lines indicate random guessing performance (0.5 for AUROC and *C*-index, 0.14 for AP). **E–G**, show the Kaplan–Meier survival estimates in low- and high-risk groups identified by the best performing model in each category (combined, EMR only and radiomics), respectively. Test set patients were stratified into two groups based on the predicted 2-year event probability at 0.5 threshold. In each case, there were significant differences in survival between the predicted risk groups (HR, 8.64, 5.96, and 4.50, respectively, *p* < 10_−18_ for all).

Twelve crowd-sourced models were developed by independent investigators during an institutional challenge over 2 months by leveraging EMR and imaging data. These models can be broadly classified as using EMR factors only, imaging only, or combining all data sources (Fig. 2A; Table 1; for detailed descriptions, see Supplementary Materials and Methods). In addition to the required 2-year event probabilities, 10 models included lifetime risk predictions and seven included the full predicted survival curves. All models performed significantly better than random on all performance measures (*p* < 0.0001 by permutation test). The top model performed significantly better in terms of AUROC than every other model (FDR < 5%), except the second-best (FDR > 5%). Most participants who used the imaging data relied on convolutional neural networks (convnets) to automatically learn predictive representations; only two combined and one radiomics-only model used handcrafted features (Fig. 2A). Of the convnets models, two of three relied on three-dimensional (3D) convolution operations (Fig. 2A). Although all EMR-only approaches used the same input data, there was significant variation in the kind of model used (linear and nonlinear, binary classifiers, proportional hazards and multitask models; Fig. 2A). The combined models used EMR data together with either tumor volume (*n* = 2), engineered radiomics (*n* = 1) or deep learning (*n* = 3).

**TABLE 1:** Summary of challenge submissions and performance metrics. All models achieved performance better than random (FDR < 5%)

Rank	Description	AUROC	AP	*C*-index
1	Deep multitask logistic regression using EMR features and tumor volume.	0.823 [0.777–0.866]	0.505 [0.420–0.602]	0.801 [0.757–0.842]
2	Fuzzy logistic regression (binary) and Cox proportional hazards model (risk prediction) using EMR features and tumor volume.	0.816 [0.767–0.860]	0.502 [0.418–0.598]	0.746 [0.700–0.788]
3	Fuzzy logistic regression (binary) or Cox proportional hazards model (risk prediction) using EMR features and engineered radiomic features.	0.808 [0.758–0.856]	0.490 [0.406–0.583]	0.748 [0.703–0.792]
4	Multitask logistic regression using EMR features.	0.798 [0.748–0.845]	0.429 [0.356–0.530]	0.785 [0.740–0.827]
5	3D convnet using cropped image patch around the tumor with EMR features concatenated before binary classification layer.	0.786 [0.734–0.837]	0.420 [0.347–0.525]	0.774 [0.725–0.819]
6	2D convnet using largest GTV image and contour slices with EMR features concatenated after additional nonlinear encoding before binary classification layer.	0.783 [0.730–0.834]	0.438 [0.360–0.540]	0.773 [0.724–0.820]
7	3D DenseNet using cropped image patch around the tumor with EMR features concatenated before multitask prediction layer.	0.780 [0.733–0.824]	0.353 [0.290–0.440]	0.781 [0.740–0.819]
8	Multilayer perceptron (MLP) with SELU activation and binary output layer using EMR features.	0.779 [0.721–0.832]	0.415 [0.343–0.519]	0.768 [0.714–0.817]
9	Two-stream 3D DenseNet with multitask prediction layer using cropped patch around the tumor and additional downsampled context patch.	0.766 [0.718–0.811]	0.311 [0.260–0.391]	0.748 [0.703–0.790]
10	2D convnet using largest GTV image and contour slices and binary output layer.	0.735 [0.677–0.792]	0.357 [0.289–0.455]	0.722 [0.667–0.774]
11	3D convnet using cropped image patch around the tumor and binary output layer.	0.717 [0.661–0.770]	0.268 [0.225–0.339]	0.706 [0.653–0.756]
12	Fuzzy logistic regression (binary) and Cox proportional hazards model (risk prediction) using engineered radiomic features.	0.716 [0.655–0.772]	0.341 [0.272–0.433]	0.695 [0.638–0.749]

### Deep Learning Using Imaging Only Achieves Good Performance and Outperforms Engineered Radiomics

Among the radiomics-only models, deep learning–based approaches performed better than hand-engineered features. In particular, nearly all deep learning models (except one) outperformed baseline-radiomics and all other models (Model 12; Fig. 2) in the binary prediction task (the smaller differences in *C*-index can be explained by the fact that most of the deep models were designed for binary classification only, and we used their binary predictions as a proxy for lifetime risk scores). Although our results indicate that deep learning models yield better performance than models relying solely on hand-engineered radiomic features, this observation, however, the increasing number of radiomics toolkits keep increasing and they offer a wealth of feature types and configuration options (27) prevent us from drawing a definitive conclusion. Nevertheless, our results show that a carefully-tuned DL model can learn features with superior discriminative power given a sufficiently large dataset.

The convnet-based models show varying levels of performance, most likely due to differences in architectures and prior image processing. Notably, the best 3D architecture (Model 9) achieves superior performance to the two-dimensional (2D) VGGNet (number 10). It incorporates several innovative features, including dense connectivity (40, 41), two-stream architecture with a downsampled context window around the tumor and a dedicated survival prediction head (detailed description in Supplementary Materials and Methods).

### EMR Features Show Better Prognostic Value than Deep Learning, Even in Combination

While imaging features can lead to strong prognostic models, the small performance gap between EMR and combined models using deep radiomics (submissions 4, 6, 7) in most cases suggests the models do not learn complementary image representations and that the performance is driven primarily by the EMR features (Fig. 2). Although one combined model using engineered features (number 3) achieved good performance, it performed worse than the exact same model using EMR features and volume only (number 2), indicating that the added complexity of radiomic features reduces performance, and the engineered features were not strong predictors on their own. Moreover, none of the radiomics-only models performed better than any of the EMR-only models (although one convnet did outperform baseline-clinical). A possible explanation is suboptimal model design that fails to exploit the complementarity between the data sources. All of the deep learning solutions incorporated EMR features in an *ad hoc* fashion by concatenating them with the image representation vector and passing them to the final classification layer. While this approach is widely used, it is not clear that it is optimal in this context. More sophisticated methods of incorporating additional patient-level information, such as for example, joint latent spaces (42) should be explored in future research.

### Impact of Volume Dependence on Model Performance

**FIGURE 3:**
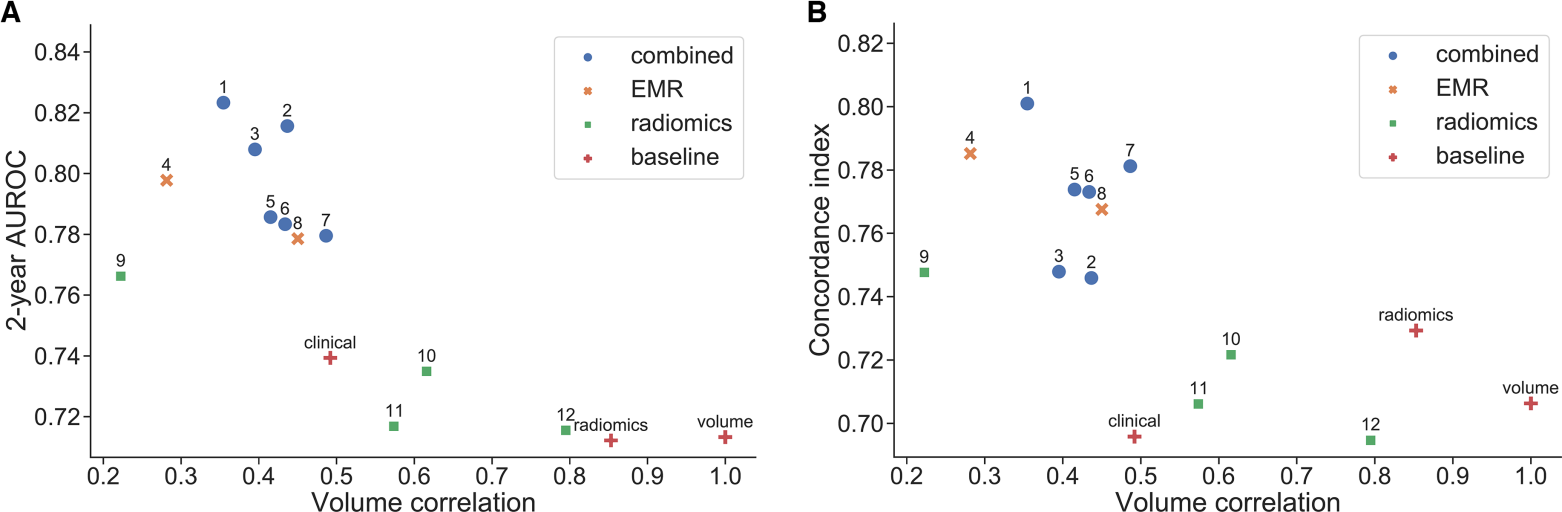
Volume dependence of predictions. Spearman rank correlation of the predictions of each model with tumor volume against performance in terms of AUROC (**A**) and *C*-index (**B**), respectively. The top models fall into an optimal region of low (but nonzero) volume correlation and high performance. Note that while models 1 and 2 used tumor volume as one of the input variables, their predictions correlate with volume only moderately (ρ < 0.5), indicating they are able to exploit additional information present in the EMR features. Higher correlation leads to decreased performance as the predictions are increasingly driven by volume only. Most radiomics-only models fall in the high correlation region (ρ ≥ 0.5), although deep learning predictions correlate at notably lower level than engineered features. Interestingly, the best radiomics submission (number 9) achieves the lowest volume correlation, suggesting that it might be using volume-independent imaging characteristics.

Recent literature has demonstrated that many radiomic signatures show strong dependence on tumor volume (29, 30), which is a simple image-derived feature and a known prognostic factor in HNC (43). We evaluated the correlation of all binary predictions with volume using Spearman rank correlation (Fig. 3). Both the baseline radiomics model and the submission using handcrafted features show high correlation (Spearman ρ = 0.79 and ρ = 0.85, respectively), suggesting that their predictions are driven primarily by volume dependency. The predictions of two out of three convnets also show moderate to strong correlation with tumor volume, albeit smaller than engineered features (ρ > 0.5). Interestingly, predictions of the best radiomics-only model (number 9) show only weak correlation with volume (ρ = 0.22) and are more discriminative than volume alone (AUROC = 0.77), suggesting that it might be possible to learn volume-independent image-based predictors.

### Top Performing Model: Multitask Learning with Simple Image Features and EMR Data

**FIGURE 4:**
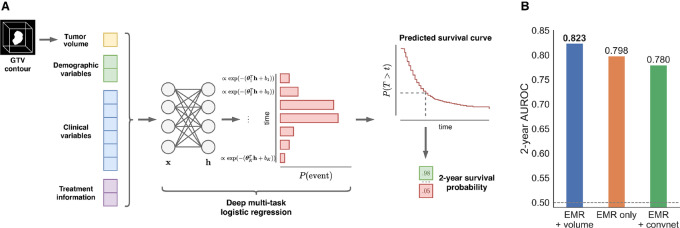
Top performing model. **A,** Overview of Deep MTLR. The model combines EMR features with tumor volume using a neural network and learns to jointly predict the probability of death at all intervals on the discretized time axis, allowing it to achieve good performance in both the binarized and lifetime risk prediction tasks. A predicted survival curve can be constructed for each individual to determine the survival probability at any timepoint. **B,** Importance of combined input data for performance on the binary endpoint. Training the deep MTLR on EMR features only led to notably worse performance. Furthermore, using a deep convolutional neural network in place of tumor volume did not improve the 2-year AUROC.

The top performing model (number 1) combined EMR data with a simple image-derived measure, and used a ML model tailored to survival prediction; a schematic overview of the submission is shown in Fig. 4. The approach is based on multitask logistic regression (MTLR), first proposed by Yu and colleagues (44) In contrast with other approaches, which focused on the binary endpoint only, MTLR is able to exploit time-to-event information by fitting a sequence of dependent logistic regression models to each interval on a discretized time axis, effectively learning to predict a complete survival curve for each patient in multitask fashion. By making no restrictive assumptions about the form of the survival function, the model is able to learn flexible relations between covariates and event probability that are potentially time-varying and nonproportional. We note that many prognostic models in clinical and radiomics literature use proportional hazards (PH) models (35, 45); however, this ignores the potential time-varying effect of features which MTLR is able to learn. Notably, when compared with the second-best model (which relies on a PH model) it achieves superior performance for lifetime risk prediction (*C* = 0.801 vs. 0.746). The added flexibility and information-sharing capacity of multitasking also enables MTLR to outperform other models on the binary task (AUROC = 0.823, AP = 0.505), even though it is not explicitly trained to maximize predictive performance at 2 years; the predicted probabilities are also better calibrated (Supplementary Fig. S2). The top performing model relies on high-level EMR features which are widely-used, easy to interpret and show strong univariate association with survival (see Supplementary Materials and Methods). The research partner incorporated nonlinear interactions by passing the features through a single-layer neural network with exponential linear unit activation (46, 47), which resulted in better performance in the development stage. The only image-derived feature used is primary tumor volume, a known prognostic factor in HNC. Using EMR features only, led to a decrease in performance (AUROC = 0.798, AP = 0.429), as did replacing tumor volume with deep image representations learned by a 3D convnet (AUROC = 0.766; Fig. 4A).

### External Validation of Best Performing Models

**FIGURE 5:**
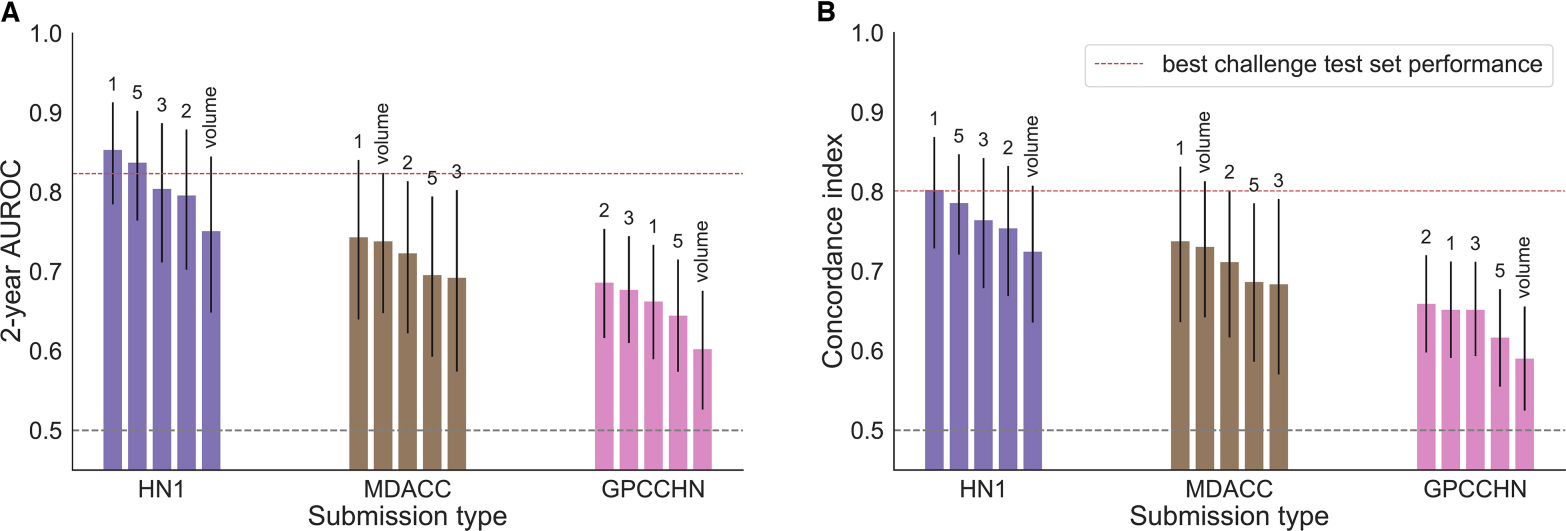
External validation performance of the top three submissions and the best deep learning submission (number 5): AUROC (**A**) and *C*-index (**B**). The red line indicates the performance achieved by the winning submission in the challenge test set. The performance of tumor volume alone is shown for comparison.

To evaluate the capacity of all the models to generalize to new patient populations, we tested their performance on three external HNC datasets (Figs. 1B and 5). There were significant differences in the distributions of several clinical and demographic variables between the challenge test set and the external datasets, most notably disease site and HPV status, as well as the target outcome prevalence (Supplementary Table S1). In line with the significant distribution shift, we observed a drop in performance in two out of three datasets, although the performance remained significantly better than random (*p* < 0.0001 for all models’ performance by permutation test). However, the top performing model maintained its rank in all datasets except GPCCHN, where it was outperformed by the simpler linear model using EMR features and volume (model number 2). The overall ranking was fairly consistent between datasets, with the exception of HN1 where engineered radiomics and deep learning combined with EMR features achieved higher performance than in the original test set (model numbers 3 and 5); however, they did not outperform the top performing model using EMR features and tumor volume. All validated models performed better than tumor volume alone, with the exception of the MDACC dataset where only the top performing model outperformed volume alone by a small margin.

## Discussion

The ability to estimate a patient's future disease progression is a critical precursor to personalized medicine. This prediction guides treatment decisions, as well as difficult personal decisions regarding risks and benefits of intensive treatment; AI and ML have the potential to assist in this important clinical decision making paradigm. However, it is not yet clear from published studies whether this potential to assist will translate to changes in clinical decision making due to limited abilities to assess generalizability of models. In this work, the different academic backgrounds and computational approaches used by our collaborating partners resulted in a diverse collection of models to evaluate. In addition, our multisite external validation of the top performing models provided insights into the importance of external evaluation and understanding differences in patient populations.

The best individual approach achieved strong performance on both 2-year and lifetime risk prediction using a multitask survival modeling framework. This demonstrates the benefit of using a flexible approach designed specifically for the task of survival prediction. In addition, because the approach relies on widely used and easy-to-interpret features (e.g., tumor stage, volume), it is attractive from a clinical standpoint as a risk stratification and monitoring tool. The model predictions are highly significant, even when adjusted for disease site, demonstrating the potential of learning from large cross-sectional datasets, as opposed to highly curated patient subsets (which has been the dominant paradigm thus far). However, it should also be mentioned that the selection of modeling methods and model inputs were determined by independent investigators during an institutional challenge, therefore it is feasible that more highly performant strategies exist and were not considered in this study.

We further assessed the generalizability of the best prognostic models using independent patient cohorts from three different institutions. In this validation phase, the top performing model retained its winning rank in two of the three datasets. The observed differences in performance help us investigate the generalizability of our models. We hypothesize that the decrease in performance is due to a distribution shift in clinical and demographic characteristics, as well as in image acquisition parameters (particularly for models 3 and 5). In particular, the GPCCHN dataset has a disproportionately high number of HPV− patients compared to the training dataset (Supplementary Table S1). This finding of limited generalizability has significant implications for the use of clinical trial data in the development of predictive models or in the validation of models within the highly selected clinical trials population as these trial populations may have significant variations from routine clinical practice (48, 49).

This is important to highlight because a model's clinical utility is not defined solely by its geographical generalizability (50). Furthermore, our external validation experiments also tested the reusability of our models and code. Through interinstitutional collaborations, our code was applied to both public and private datasets by individuals not involved in development, demonstrating the accessibility of our methodologies and motivating utilization of open science for scrutiny and advancement of scientific results. Toward this end, we have also made our large training set available for scientific scrutiny and collaboration via The Cancer Imaging Archive (37) which allows access to both the imaging and associated clinical variables used in this project. We would encourage all future efforts to continue to share as much data as is feasible to ensure reproducibility within the field.

The utility of radiomics in HNC survival prediction has been investigated in recent studies (13, 15, 16). We have identified several strong radiomics predictors; however, the best performing individual model used EMR features, with primary tumor volume as the only image-derived feature. Our conclusions match those of Ger and colleagues (13), who did not find significant improvement in prognostic performance of handcrafted CT and PET imaging features in HNC compared with volume alone and of Vallières and colleagues (15), whose best performing model for overall survival also combined EMR features and volume. We further showed that although deep learning–based imaging models generally outperformed approaches based on handcrafted features, none proved superior to the combined EMR-volume model, even when combined with EMR data. Deep learning methods achieve excellent performance in many image processing tasks (51); however, current approaches require substantial amounts of training data. The endpoint contribution of individual data modalities can be characterized by comparing the model's performance with the baseline EMR, volume, and radiomics models (Fig. 2). By comparing individual baseline models with developed models, we can see how different model designs contribute to predictive performance. The contribution of each modality and their combined improvements can be seen in MTLR's performance across various inputs (Fig. 4B).

While our training dataset is the largest publicly-available single-institution HCN imaging collections, it is still relatively small compared with natural image datasets used in ML research, which often contain millions of samples (52). Although such large sample sizes might be infeasible in a medical context, better data collection and sharing practices can help build more useful databases [the UK Biobank (53) or The Cancer Genome Atlas (54) are excellent examples]. This is especially important in diseases with low event rates, where a substantial number of patients might be needed to capture the variation in phenotype and outcomes. The inferior performance of radiomic models can also be attributed to suboptimal imaging data. While the possibility to easily extract retrospective patient cohorts makes routine clinical images attractive for radiomics research, they are often acquired for clinical purposes and may not be sufficiently standardized for new biomarker discovery. CT images in particular might not accurately reflect the biological tumor characteristics due to insufficient resolution, sensitivity to acquisition parameters and noise (55, 56), as well as the source of image contrast, which is essentially electron density of the tissue which demonstrates little texture at current image scales. This highlights the broader need of greater collaboration between ML researchers, clinicians, and physicists, also in data selection and experiment design—with reciprocal feedback (57, 58).

Our study has several potential limitations. Participation in the model development was restricted to researchers at one institution, which limited the number of models that could be explored. In addition, the hand-engineered radiomics submissions relied on one radiomics toolkit (PyRadiomics), while other widely-used toolkits make use of potentially different feature sets and definitions; however, thanks to recent efforts in image biomarker standardization, the features have been shown to be largely consistent between the major implementations (27). While smoking status may be considered a relevant variable for HNC prognosis, it is not guaranteed to be included in all predictive models and does not significantly improve predictive performance (Supplementary Fig. S3 and S4). Furthermore, because our study was structured as a Challenge, selection of modeling methods and input variables were left to the discretion of the participants. It is likely that more sophisticated ensembling methods [e.g., Bayesian model averaging (59) or stacking (60)] could achieve even better performance by weighing the models according to their strengths. We leave this exploration for future work.

In the future, we would like to further enhance the diversity of approaches and help us validate our conclusions by expanding our crowdsourcing efforts beyond our institution. We are also working on collecting additional outcome information, including recurrence, distant metastasis, and treatment toxicity, which would provide a richer set of prediction targets and might be more relevant from a clinical standpoint. The importance of ML and AI as tools of precision medicine will continue to grow. However, it is only through transparent and reproducible research that integrates diverse knowledge that we can begin to realize the full potential of these methods and permit integration into clinical practice. Any clinical trials that employ AI/ML models as a component of either patient selection, treatment selection, or attempt to translate models derived from clinical trials populations should be aware of intrinsic risks related to generalizability which may impact performance of those models outside the bounds of the dataspace in which the models were generated.

## Supplementary Material

Supplementary Figure S1 Patient selection and data curation process.

Supplementary Figure S2 Calibration of predicted 2-year event probabilities for the best performing model in each category and the ensemble of all models.

Supplementary Figure S3 Model performances across permutations by clinical (EMR) variables.

Supplementary Figure S4 Model performances across permutations by clinical and imaging variables.

Supplementary Data Supplementary Data of figures/tables

## Footnotes

^1^

https://wiki.cancerimagingarchive.net/display/Public/Head-Neck-Radiomics-HN1

^2^

https://wiki.cancerimagingarchive.net/display/Public/HNSCC
