# Supplementary figures and images for: Multi-institutional Prognostic Modeling in Head and Neck Cancer: Evaluating Impact and Generalizability of Deep Learning and Radiomics
Source: Cancer Res Commun. 2023 Jun 29;3(6):1140–51. doi: 10.1158/2767-9764.CRC-22-0152 (PMC10309070; doi:10.1158/2767-9764.CRC-22-0152)

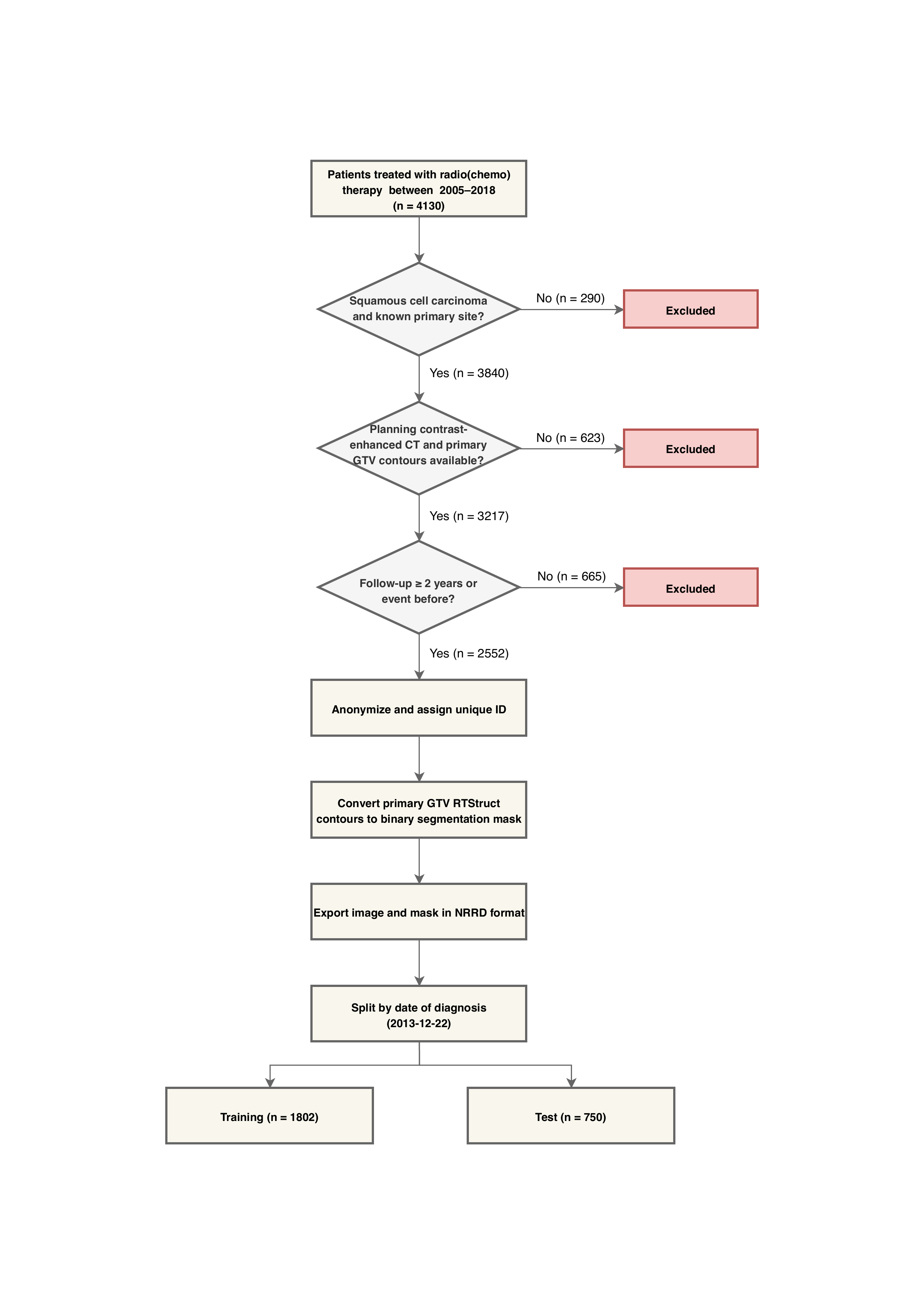

Supplement: Supplementary Figure S1 — Patient selection and data curation process. [file crc-22-0152-s01.png]

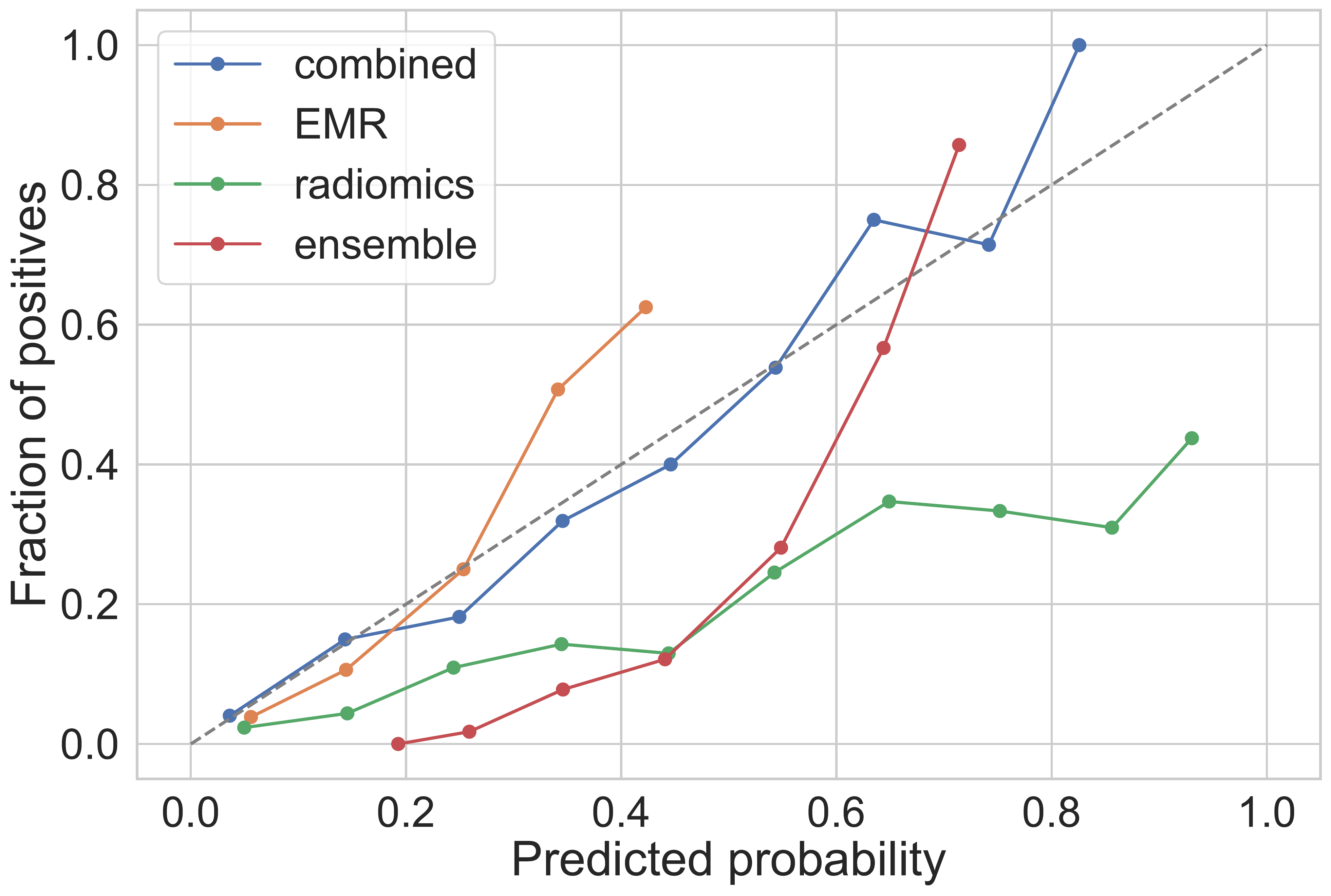

Supplement: Supplementary Figure S2 — Calibration of predicted 2-year event probabilities for the best performing model in each category and the ensemble of all models. [file crc-22-0152-s02.png]

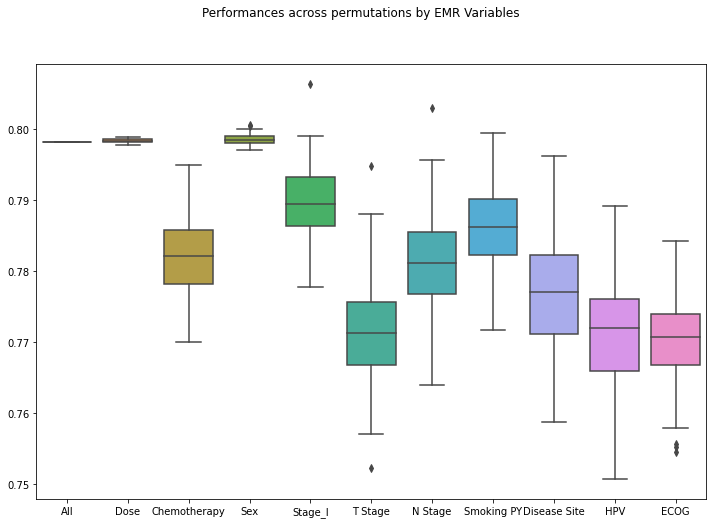

Supplement: Supplementary Figure S3 — Model performances across permutations by clinical (EMR) variables. [file crc-22-0152-s03.png]

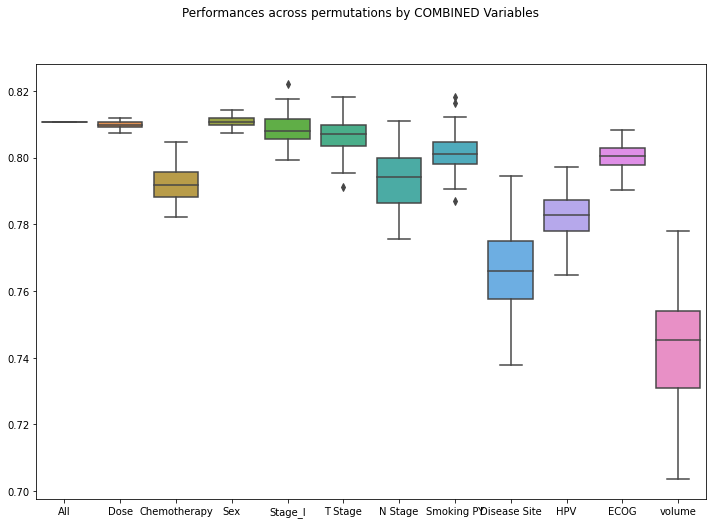

Supplement: Supplementary Figure S4 — Model performances across permutations by clinical and imaging variables. [file crc-22-0152-s04.png]
