# Supplementary material for: Multi-institutional Prognostic Modeling in Head and Neck Cancer: Evaluating Impact and Generalizability of Deep Learning and Radiomics
Source: Cancer Res Commun. 2023 Jun 29;3(6):1140–51. doi: 10.1158/2767-9764.CRC-22-0152 (PMC10309070; doi:10.1158/2767-9764.CRC-22-0152)
Supplement: Supplementary Data — of figures/tables [file crc-22-0152-s05.docx]

# Supplementary material

## Data curation and preprocessing


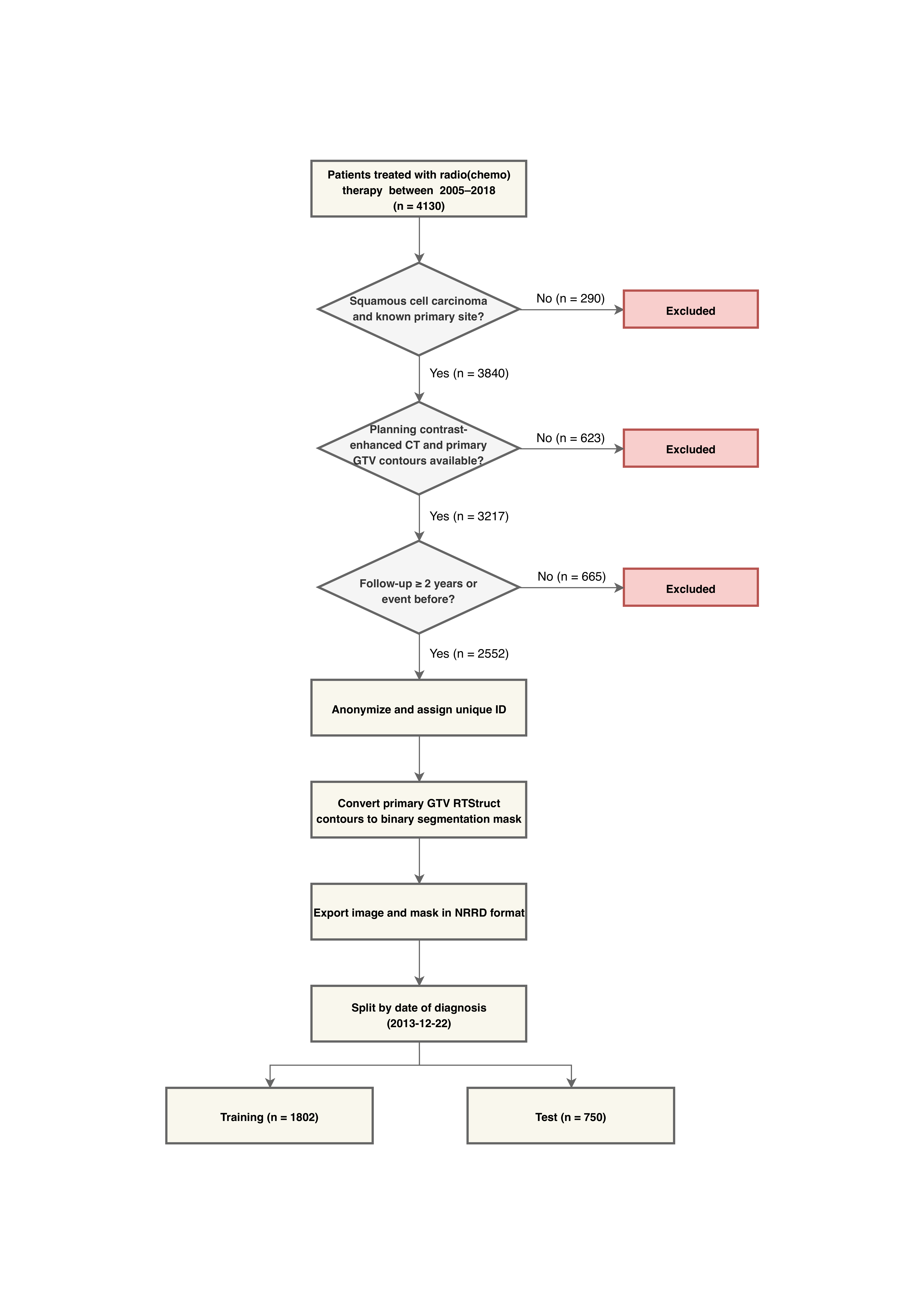


**Supplementary Figure S1.** Patient selection and data curation process.

## Patient characteristics

**Supplementary Table S1.** Patient characteristics in the training and test sets. Variables marked with a * differ significantly from the challenge test set ($p<.05$ after multiple testing correction, chi-squared test).

|  | **RADCURE** | | **HN1** | **MDACC** | **GPCCHN** |
| --- | --- | --- | --- | --- | --- |
|  | **Training** | **Test** |  |  |  |
| # of patients | 1802 | 750 | 130 | 444 | 298 |
| **Outcome** |  |  |  |  |  |
| Alive/Censored | 1065 (59%) | 609 (81%) | 60 (46%)* | 342 (77%) | 179 (60%)* |
| Dead | 737 (41%) | 141 (19%) | 70 (54%)* | 102 (23%) | 119 (40%)* |
| Dead before 2 years | 323 (18%) | 103 (14%) | 29 (22%)* | 32 (7%)* | 79 (27%)* |
| **Sex** |  |  |  |  |  |
| Male | 1424 (79%) | 615 (82%) | 105 (81%) | 380 (86%) | 222 (74%)* |
| Female | 378 (21%) | 135 (18%) | 25 (19%) | 64 (14%) | 76 (26%)* |
| **Disease Site** |  |  |  |  |  |
| Oropharynx | 777 (43%) | 399 (53%) | 85 (65%)* | 444 (100%)* | 134 (45%)* |
| Larynx | 555 (31%) | 172 (23%) | 45 (35%)* | 0 (0%)* | 86 (29%)* |
| Nasopharynx | 220 (12%) | 101 (13%) | 0 (0%)* | 0 (0%)* | 16 (5%)* |
| Hypopharynx | 115 (6.4%) | 28 (3.7%) | 0 (0%)* | 0 (0%)* | 32 (11%)* |
| Lip / Oral Cavity | 72 (4.0%) | 10 (1.3%) | 0 (0%)* | 0 (0%)* | 30 (10%)* |
| Nasal Cavity | 31 (1.7%) | 20 (2.7%) | 0 (0%)* | 0 (0%)* | 0 (0%)* |
| Paranasal Sinus | 16 (0.9%) | 10 (1.3%) | 0 (0%)* | 0 (0%)* | 0 (0%)* |
| Esophagus | 14 (0.8%) | 8 (1.1%) | 0 (0%)* | 0 (0%)* | 0 (0%)* |
| Salivary Glands | 2 (0.1%) | 2 (0.3%) | 0 (0%)* | 0 (0%)* | 0 (0%)* |
| **T stage** |  |  |  |  |  |
| 1/2 | 919 (51%) | 405 (54%) | 65 (50%) | 265 (60%)* | 110 (37%)* |
| 3/4 | 855 (47%) | 336 (45%) | 65 (50%) | 179 (40%)* | 188 (63%)* |
| Not available | 28 (2%) | 9 (1%) | 0 (0%) | 0 (0%)* | 0 (0%)* |
| **N stage** |  |  |  |  |  |
|  | 688 (38%) | 226 (30%) | 58 (45%)* | 41 (9%)* | 96 (32%) |
|  | 170 (9%) | 90 (12%) | 16 (12%)* | 48 (11%)* | 40 (13%) |
|  | 835 (46%) | 395 (53%) | 53 (41%)* | 344 (77%)* | 154 (52%) |
|  | 108 (6%) | 39 (5%) | 3 (2%)* | 11 (2%)* | 8 (3%) |
| Not available | 1 (<1%) | 0 (0%) | 0 (0%)* | 0 (0%)* | 0 (0%) |
| **AJCC stage** |  |  |  |  |  |
| I/II | 455 (25%) | 158 (21%) | 35 (27%) | 17 (4%)* | 41 (14%)* |
| III/IV | 1318 (73%) | 581 (77%) | 95 (73%) | 427 (96%)* | 257 (86%)* |
| Not available | 29 (2%) | 11 (1%) | 0 (0%) | 0 (0%)* | 0 (0%)* |
| **HPV status** |  |  |  |  |  |
| Positive | 513 (28%) | 327 (44%) | 22 (17%)* | 231 (52%)* | 29 (10%)* |
| Negative | 269 (15%) | 168 (22%) | 55 (42%)* | 42 (9%)* | 18 (6%)* |
| Not tested | 1020 (57%) | 255 (34%) | 53 (41%)* | 171 (39%)* | 251 (84%)* |
| **Systemic Therapy** |  |  |  |  |  |
| Yes | 725 (40%) | 361 (48%) | 34 (26%)* | 392 (88%)* | 239 (80%)* |
| No | 1077 (60%) | 389 (52%) | 96 (74%)* | 52 (12%)* | 59 (20%)* |
| **ECOG performance status** |  |  |  |  |  |
| 0 | 1120 (62%) | 436 (58%) | 60 (46%)* | 0 (0%)* | 80 (27%)* |
| 1 | 489 (27%) | 290 (39%) | 56 (43%)* | 0 (0%)* | 216 (72%)* |
| 2 | 145 (8%) | 20 (3%) | 3 (2%)* | 0 (0%)* | 2 (1%)* |
| >2 | 34 (2%) | 4 (1%) | 1 (1%)* | 0 (0%)* | 0 (0%)* |
| Not available | 14 (1%) | 0 (0%) | 10 (8%)* | 444 (100%)* | 0 (0%)* |

## Imaging protocols

**Supplementary Table S2.** CT imaging parameters used in the study. Images in the RADCURE dataset were acquired using either of: Toshiba Aquilion One ($n=1653$), GE Discovery ST ($n=643$), GE LightSpeed Plus ($n=130$), Philips Brilliance Big Bore ($n=96$) or GE Discovery 610 ($n=30$). Images in the GPCCHN were acquired without contrast enhancement using either of: Siemens SOMATOM Definition AS ($n=225$), Siemens Sensation Open ($n=71$) or GE Optima CT660 ($n=2$). Imaging parameters of the other publicly-available datasets have been described in the corresponding publications. ^35,36^

| Parameter | Median [range] | |
| --- | --- | --- |
|  | RADCURE | GPCCHN |
| Slice thickness | 2 [2–2.5] | 3 [2–5] |
| kVp | 120 [120–120] | 120 [100–120] |
| Exposure [mAs] | 300 [121–540] | 58 [5–538] |
| Pixel spacing [mm] | .976 [.702–1.17] | 0.977 [.939–1.56] |

## Smoking Status

Smoking has been associated with survival in head and neck cancer but multiple studies suggest that this effect is likely limited to ‘current’ smokers due to potential interactions between treatment and hypoxia induced by smoking. As such, smoking remains a relevant, but not guaranteed variable to be included in predictive models  ^61–63^.

Despite this, the authors anticipated that the inclusion of smoking status would improve the performance of the baseline model, but not the ordering of comparator models. To test this, using data updated after the completion of the challenge, we refit the baseline model including smoking status and found that the AUC of the baseline clinical model increased from 0.74 to 0.76. Although a seemingly important variable to add, it still did not outperform our best performing model, or our best performing radiomics based model.

## Detailed descriptions of models

### Model 1

Multi-task logistic regression (MTLR) uses a sequence of dependent regressors to predict the probability of event occuring at multiple discrete timepoints in a multi-task fashion. ^44^ The model is trained by minimizing the MTLR log-likelihood:

$$\begin{matrix} L\left( \boldsymbol{\Theta},D \right) & =\sum_{j=1}^{N-N_{c}-1} \sum_{k=1}^{K-1} \left( \boldsymbol{\theta}_{k}^{T}{\boldsymbol{\phi}\left( \mathbf{x} \right)}^{\left( j \right)}+b_{k} \right)y_{k}^{\left( j \right)} & \text{(Uncensored)} \\ & +\sum_{j=N-N_{c}}^{N} \log\left( \sum_{i=1}^{K-1} \mathbf{1}\{t_{i}\geq T_{c}^{\left( j \right)}\}exp\left( \sum_{k=i}^{K-1} \left( \left( \boldsymbol{\theta}_{k}^{T}{\boldsymbol{\phi}\left( \mathbf{x} \right)}^{\left( j \right)}+b_{k} \right)y_{k}^{\left( j \right)} \right) \right) \right) & \text{(Censored)} \\ & -\sum_{j=1}^{N} \log\left( \sum_{i=1}^{K} \exp\left( \sum_{k=i}^{K-1} \boldsymbol{\theta}_{k}^{T}{\boldsymbol{\phi}\left( \mathbf{x} \right)}^{\left( j \right)}+b_{k} \right) \right), & \text{(Normalizing constant)} \\ & +\frac{C_{1}}{2}\sum_{k=1}^{K-1} \|\boldsymbol{\theta}_{k}{\|}_{2}^{2} & \text{(Regularizer)}, \end{matrix}$$

where $\left( \boldsymbol{\theta}_{k}, b_{k} \right)$ are trainable parameters associated with the $k$th timepoint, $D=\{T^{\left( j \right)},\delta^{\left( j \right)},\mathbf{x}^{\left( j \right)}{\}}_{j=1}^{N}$ is a dataset with $N$ patients, $N_{c}$ of whom are censored and $y_{k}$ is the binary event indicator for timepoint $k$. We define $\phi\left( x \right)$ to be a multi-layer perceptron with exponential linear unit (ELU) activations. ^46,47^

The model takes a vector of EMR features and volume as input and predicts the probability of event occurring within each of the discrete time intervals. An individual survival curve can be computed for each patient from the predictions, from which we read out the probability of 2-year survival, as well as the lifetime risk score. Tumour volume was computed using the mesh algorithm implemented in PyRadiomics version 2.2.0. We used one-hot encoding for categorical EMR features and encoded any missing values as separate dummy category. The inputs were normalized to zero mean and unit variance using statistics computed on the training set. We implemented the MTLR algorithm using using the PyTorch framework version 1.5.0 and trained it for 100 epochs using the Adam optimizer with default momentum parameters. ^64^ The number of MTLR time bins was set as $\sqrt{N_{\mathrm{uncensored}}}$, where $N_{\mathrm{uncensored}}$ is the number of uncensored patients in the training set and the bin edges were set to quantiles of training survival time distribution. We tuned other hyperparameters by maximizing 5-fold cross validation AUROC on the training set using 60 iterations of random search. The final hyperparameter settings are shown in the table below. Code link: <https://github.com/bhklab/uhn-radcure-challenge/tree/master/submissions/5e515f3-c22638c>

**Supplementary Table S3.** Submission 1 hyperparameters.

| Hyperparameter | Value |
| --- | --- |
| Batch size | 512 |
| Dropout | .24 |
| Hidden layer sizes | (128) |
| $C_{1}$ | 10 |
| Learning rate | .006 |
| Weight decay | $6\times{10}^{-5}$ |

### Model 2

We attempted to identify prognostic signal in EMR variables using a fuzzy learning approach. ^65^ Briefly, we divided the training dataset into 2 groups based on the tumour volume (greater/less than median) and trained a binary logistic regression model to predict the probability of falling in the large volume group from the input features. We then built a separate logistic regression (for the binary task) and Cox proportional hazard (for the lifetime survival task) within each of the subgroups. The final predictions were computed as a linear combination of the subgroup model predictions weighted by the predicted probability of falling within the subgroup. We used the provided EMR features as inputs. All categorical features were converted to indicator variables (with a separate category for missing values) and continuous features were normalized to zero mean and unit variance. Code link: <https://github.com/bhklab/uhn-radcure-challenge/tree/master/submissions/c927742-c985b3d-5af6c56>

### Model 3

We attempted to identify prognostic signal in radiomic features beyond tumour volume using the fuzzy learning approach described in Model 2. ^65^ We used the provided EMR features as inputs together with hand-engineered imaging features. To reduce the dimensionality of feature space and prevent the inclusion of potentially noisy variables, we used a curated set of radiomic features which were previously found to be prognostic in HNC: GLSZM-SizeZoneNonUniformity, GLSZM-ZoneVariance, GLRLM-LongRunHighGrayLevelEmphasis^15,16^. We extracted the features using PyRadiomics 2.2.0 with fixed quantization bin width equal to 25 and after resampling the images to isotropic 1mm voxel spacing. Standard logistic regression (as implemented in Scikit-learn package ^66^, version 0.22.1) with LBFGS solver and inverse frequency weighted loss function was used for binarized output, Cox modeling (Lifelines package, version 0.24.5) with partial hazard fitting and step size of 0.5 was used for survival prediction. Code link: <https://github.com/bhklab/uhn-radcure-challenge/tree/master/submissions/c927742-c985b3d-5af6c56>

### Model 4

The same algorithm as Model 1 using only EMR data and different hyperparameter settings tuned using 5-fold cross validation (Table [S4](#tab:sub4_hparams)). Code link: <https://github.com/bhklab/uhn-radcure-challenge/tree/master/submissions/5e515f3-c22638c>

**Supplementary Table S4.** Hyperparameters of submission 4.

| Hyperparameter | Value |
| --- | --- |
| Batch size | 1024 |
| Dropout | .14 |
| Hidden layer sizes | (32, 32, 32) |
| $C_{1}$ | 10 |
| Learning rate | .006 |
| Weight decay | $1.3\times{10}^{-6}$ |

### Model 5

Our approach uses a 3D convnet with EMR features concatenated before fully-connected layers. The convnet uses conv-batch norm-leaky ReLU block structure with negative activation slope equal to .1. The network takes a $50mm\times50mm\times50\mathrm{mm}$ image patch centred on the GTV mask centroid as input and outputs the predicted probability of death before 2 years. The images were resampled to isotropic 1mm spacing, intensity clipped to [-500 HU, 1000 HU] range and normalized by subtracting the training set mean and dividing by training standard deviation. EMR features were normalized analogously and categorical features were additionally one-hot encoded, replacing any missing values with a special ’missing’ category. We applied random data augmentation, including random in-plane rotations between $\left( -\pi/6,\pi/6 \right)$ radians, random flipping along the $x$ and $z$ axes and additive Gaussian noise with standard deviation .05 (after normalization). We implemented the model using PyTorch 1.5.0 and PyTorch Lightning 0.7.6 and trained it for 500 epochs using the Adam algorithm with batch size 10 and learning rate ${10}^{-3}$ decayed by a factor of 10 after 60, 160 and 360 epochs. To prevent overfitting, we used dropout with probability .4 and weight decay regularization with coefficient ${10}^{-4}$. We used binary cross entropy loss weighted by the inverse frequency of positive label to reduce the impact of class imbalance. The hyperparameters were selected based on performance on a 10% validation set held out from the training set. Code link: <https://github.com/bhklab/uhn-radcure-challenge/tree/master/submissions/e943420-4e7d2ea>

**Supplementary Table S5.** Convnet architecture. Each convolution operation corresponds to the block described above.

| Operation | Output channels | Kernel size |
| --- | --- | --- |
| Convolution | 64 | $5^{3}$ |
| Convolution | 128 | $3^{3}$ |
| Max pooling (stride=2) | - | $2^{3}$ |
| Convolution | 256 | $3^{3}$ |
| Convolution | 512 | $3^{3}$ |
| Max pooling (stride=2) | - | $2^{3}$ |
| Global average pooling | - | - |
| Fully-connected | 512 | - |
| Concatenate EMR features | - | - |
| Fully-connected | 512 | - |
| Dropout (p=.4) | - | - |
| Fully-connected | 1 | - |

### Model 6

We used a 2D convnet with the same architecture as Model 10 and combined the learned image features with EMR variables. The EMR features were one-hot encoded and normalized to zero mean and unit variance before being passed through three fully-connected layers with 8 hidden units and concatenated with the convnet output before the final classification layer.

### Model 7

To learn prognostic image representations, we used a 3D dense convolutional network (DenseNet) with multitask learning prediction head. The network takes a cropped $60mm\times60mm\times60\mathrm{mm}$ image patch, centred on the GTV centroid and outputs the probability of death at multiple discrete time intervals. We used convolutional block structure previously validated in retinal tomography scans. ^41^ Each convolutional block consists of multiple layers of within-slice ($1\times3\times3$) and across-slice ($3\times1\times1$) convolutions, followed by batch normalization ^67^ and ReLU nonlinearities. The EMR features were concatenated with the convnet output before the final prediction layer. The convnet architecture is shown in Table [S6](#tab:densenet) below.

**Supplementary Table S6.** 3D Dense Net architecture. The convolution kernel sizes are given as (depth, width, height). Each conv operation above corresponds to the sequence batch norm-ReLU-conv, except for the first 2 convolutions where the order is reversed. Additionally, we applied dropout after each transition layer. Note that the number of channels in each layer is determined by the first convolution output channels (here 32) and the growth rate (tunable hyperparameter).

| Block | Operations |
| --- | --- |
| Convolution |  |
| 1 |  |
| Pooling | max pool $2\times2\times2$, stride 2 |
| Dense block 1 |  |
| 1 |  |
| 3 |  |
|  | conv $1\times1\times1$ |
|  | max pool $2\times2\times2$, stride 2 |
| Dense block 2 |  |
| 1 |  |
| 3 |  |
|  | conv $1\times1\times1$ |
|  | max pool $2\times2\times2$, stride 2 |
| Dense block 3 |  |
| 1 |  |
| 3 |  |
|  | conv $1\times1\times1$ |
|  | max pool $2\times2\times2$, stride 2 |
| Dense block 4 |  |
| 1 |  |
| 3 |  |
| Global pool | adaptive average pool |
| EMR features | concat(input, EMR features) |
| Output | MTLR(time bins=40) |

The input image patches were resampled to $3mm\times1mm\times1\mathrm{mm}$ voxel spacing and clipped to range [-500, 1000] HU. For EMR features, we one-hot encoded categorical inputs features, with any missing values represented as separate category. Both images and EMR features were normalized to zero mean and unit variance using statistics computed on the training set. 10% of training patients were set aside as a validation set for hyperparameter tuning. We applied random data augmentation to input image patches (Table [S7](#tab:augmentation)). The augmentation operations were implemented in SimpleITK version 1.2.4 and fused into a single transform to minimize interpolation artifacts.

**Supplementary Table S7.** Data augmentation operations used during training. Parameter values were drawn randomly for each input from the ranges shown above.

| Parameter | Value or range |
| --- | --- |
| In-plane rotation | [-10–10] |
| Flip along z-axis | [True, False] |
| In-plane shear | [-.005, .005] |
| In-plane scaling | [.8, 1.2] |
| In-plane translation | [-10mm, 10mm] |
| In-plane elastic deformation | $\text{grid size}=\left( 2,2 \right)$, $\alpha=5$ |
| Gaussian noise | $\mu=0$, $\sigma=10$ |

We implemented our approach using PyTorch version 1.5.0 and PyTorch Lightning version 0.7.6. The model was trained by minimizing the MTLR negative log likelihood using the Adam optimizer with default momentum parameters for a maximum of 200 epochs. Training was stopped early if the loss on the validation set did not improve by at least .0005 for 20 epochs. The initial learning rate was decayed by a factor of .5 after 60, 100, 140 and 180 epochs. To mitigate the high class imbalance present in the dataset, we oversampled the minority class by using a balanced minibatch sampler, which helped to stabilize training. Hyperparameters were selected by maximizing the validation set performance using 60 iterations of random search. The final hyperparameter configuration is shown below. Code link: <https://github.com/bhklab/uhn-radcure-challenge/tree/master/submissions/a4c8caa-60f36db>

**Supplementary Table S8.** Submission 7 hyperparameters.

| Hyperparameter | Value |
| --- | --- |
| Batch size | 8 |
| Dense block layers | (2, 2, 2, 3) |
| DenseNet growth rate | 24 |
| Dropout | .38 |
| Initial num. channels | 32 |
| MTLR regularization | 10 |
| Learning rate | .0002 |
| Weight decay | $9.4\times{10}^{-4}$ |

### Model 8

We trained a three-layer neural network with scaled exponential linear unit (SELU) activation and alpha-dropout. ^68^ The inputs were EMR features after one-hot encoding (for categorical features) and normalization (for continuous features) and the output was the predicted 2-year survival probability. To address the issue of class imbalance, we used biased sampling to adjust the frequency of positive training samples. We selected the hyperparameters manually based on cross-validation performance.

### Model 9

We used a 3D dense convolutional network (DenseNet) with similar block structure and architecture as Model 7. The main differences were the lack of EMR inputs and a second context network, taking a downsampled image patch with the same location as the base network but $2\times$ lower resolution, providing a zoomed-out view of the tumour surroundings. The feature maps from both streams were concatenated along the channel dimension before global pooling and passed through additional $1\times1\times1$ convolution to maintain equal number of channels. The final hyperparameter configuration is shown below. Code link: https://github.com/bhklab/uhn-radcure-challenge/tree/master/submissions/a4c8caa-60f36db

**Supplementary Table S9.** Submission 9 hyperparameters.

| Hyperparameter | Value |
| --- | --- |
| Batch size | 16 |
| Dense block layers | (2, 2, 2, 3) |
| DenseNet growth rate | 24 |
| Dropout | .03 |
| Initial num. channels | 32 |
| MTLR regularization | 10 |
| Learning rate | .00027 |
| Weight decay | $1.7\times{10}^{-4}$ |

### Model 10

The architecture used was a VGGNet ^69^ with batch normalization and sigmoid output for binary classification. The inputs were formed by extracting the largest 2D GTV slice and concatenating with the binary mask along the channel axis. The images were cropped to $96\times96$ pixel window centred on the mask centroid, intensity clipped to [-1000, 400] HU range and normalized to zero mean and unit variance. We applied data augmentation including additive Gaussian noise (image channel only) with standard deviation of 12 HU, random translations between $\pm20$ pixels in each direction and random scaling between .85 and 1.25. The model was implemented in PyTorch and trained with minority class oversampling to mitigate class imbalance. We used the reference implementation of VGGNet and pretrained weights available in the torchvision package version 0.6.0.

### Model 11

We used similar training setup and implementation as submission 5 but relying on images only (without EMR features) and a different convnet architecture (see below). Code link: <https://github.com/bhklab/uhn-radcure-challenge/tree/master/submissions/e943420-4e7d2ea>

**Supplementary Table S10.** Convnet architecture.

| Operation | Output channels | Kernel size |
| --- | --- | --- |
| Convolution | 64 | $3^{3}$ |
| Convolution | 128 | $3^{3}$ |
| Convolution | 128 | $3^{3}$ |
| Max pooling (stride=2) | - | $2^{3}$ |
| Convolution | 256 | $3^{3}$ |
| Convolution | 256 | $3^{3}$ |
| Convolution | 512 | $3^{3}$ |
| Max pooling (stride=2) | - | $2^{3}$ |
| Convolution | 512 | $3^{3}$ |
| Convolution | 1024 | $3^{3}$ |
| Convolution | 1024 | $3^{3}$ |
| Max pooling (stride=2) | - | $2^{3}$ |
| Global average pooling | - | - |
| Fully-connected | 1024 | - |
| Dropout (p=.4) | - | - |
| Fully-connected | 1 | - |

### Model 12

We applied the fuzzy training framework used in submissions 2 and 3 to the curated set of radiomic features only: GLSZM-SizeZoneNonUniformity, GLSZM-ZoneVariance, GLRLM-LongRunHighGrayLevelEmphasis and tumour volume. Code link: <https://github.com/bhklab/uhn-radcure-challenge/tree/master/submissions/c927742-c985b3d-5af6c56>

## Additional results


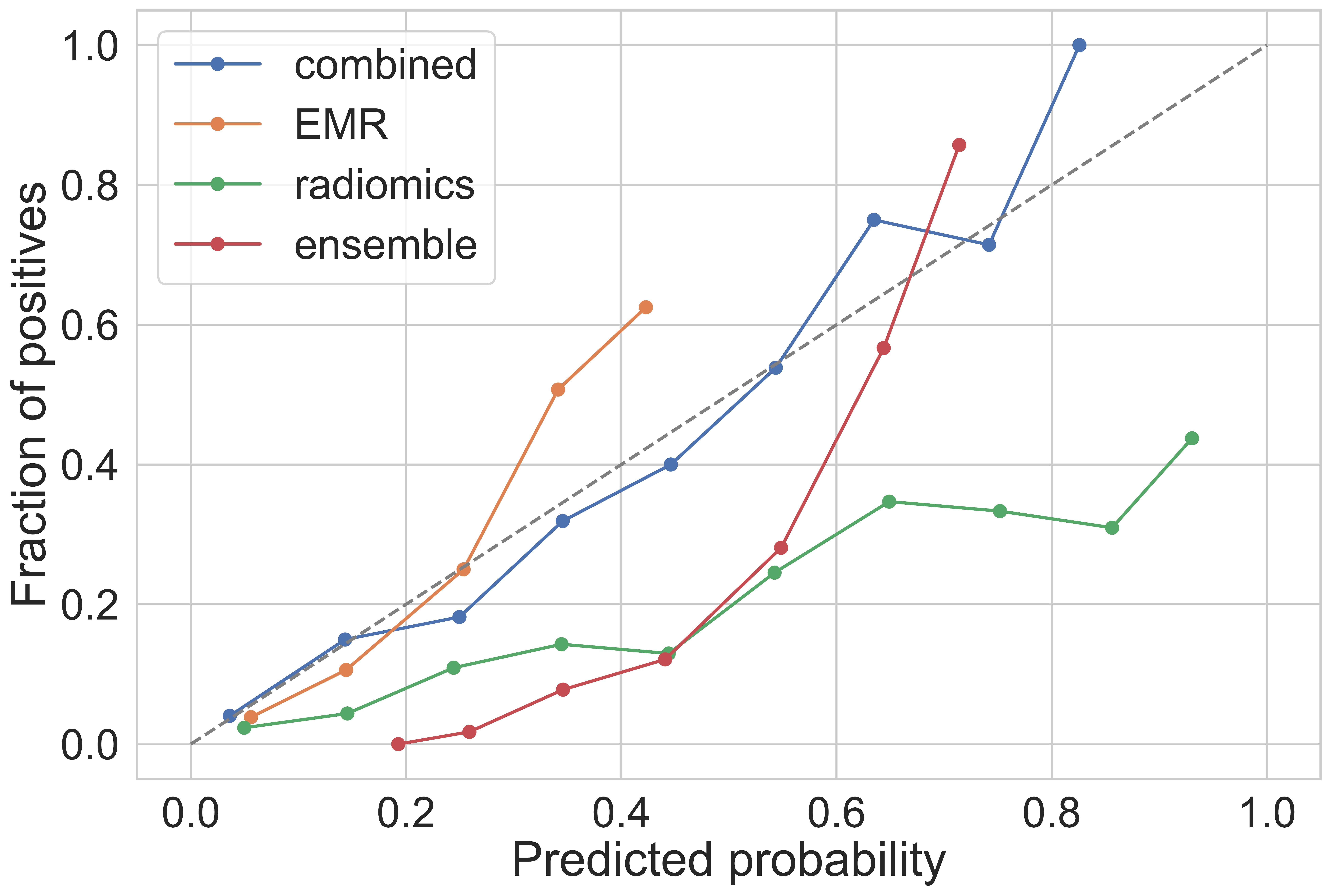


**Supplementary Figure S2.** Calibration of predicted 2-year event probabilities for the best performing model in each category and the ensemble of all models.

## Exploration of input data contribution

To explore the contribution of each clinical (EMR) variable in the best-performing model, we performed a permutation-based analysis showing the loss of prognostic value when each variable is replaced by random Gaussian noise (Supplementary Figures [3](#fig:var-contr-A) and [4](#fig:var-contr-B)). As smoking has been associated with survival in head and neck cancer  ^61–63^, we added it into the comparison despite its unavailability for all our validation sets.

The permutation analysis shows the amount of performance loss when each variable is replaced by random Gaussian noise. (A) Change in performance when each clinical variable is randomized. A variable’s degree of contribution to the model’s predictive power can be estimated by the amount of decreased performance. (B) Same analysis with combining clinical and imaging variables. As expected, tumor volume is the greatest contributing variable to model performance.


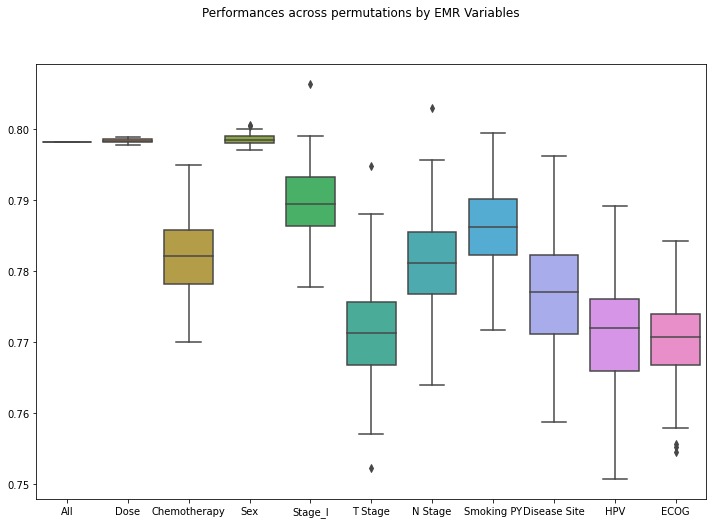


**Supplementary Figure S3.** Model performances across permutations by clinical (EMR) variables.


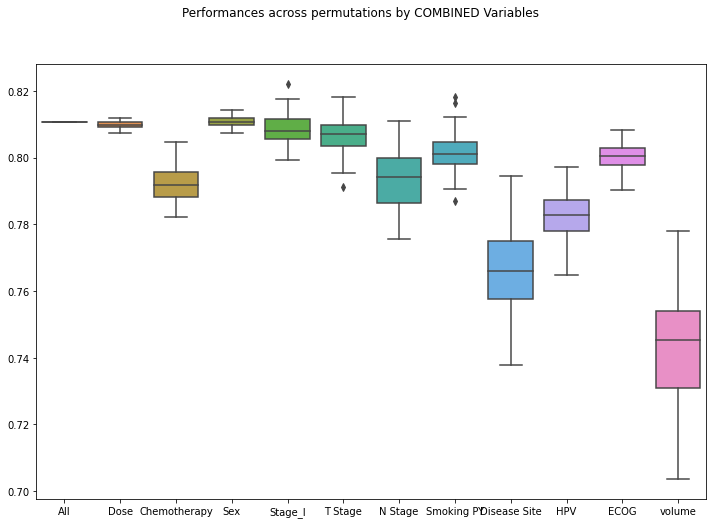


**Supplementary Figure S4.** Model performances across permutations by clinical and imaging variables.
